# Outcomes of nonsurgical endodontic treatment under general anesthesia in special health care needs: An observational study

**DOI:** 10.1038/s41405-024-00224-5

**Published:** 2024-05-21

**Authors:** Fahd Alsalleeh, Fatima Y. Albishry, Asrar S. Aleyiydi, Farah S. Aldossari, Norah H. Alharbi, Maha Alghofaily, Riyadh Althumairy

**Affiliations:** 1 Restorative Dental Sciences, College of Dentistry, King Saud University, Riyadh, Saudi Arabia; 2 King Saud University Medical City - Dental University Hospital, King Saud University, Riyadh, Saudi Arabia

**Keywords:** Endodontics, Root canal treatment

## Abstract

**Background and aim:**

The alarming rise in the number of people with special health care needs (SHCNs) necessitates a paradigm shift in how to approach their oral health needs. General anesthesia (GA) is a valuable technique for facilitating dental procedures in patients with SHCNs who may not be able to tolerate treatment without it. The aim was to assess nonsurgical endodontic treatment and outcomes in patients with SHCNs performed under GA.

**Method:**

Seventy-eight permanent teeth in 33 patients who received nonsurgical endodontic treatment under GA were included between 2018 and 2022 in SHCNs hospital clinics. The demographic data, types of SHCNs, pulpal and periapical diagnosis, type of treatment, and material used were analyzed. All patients were recalled for clinical and radiographic examinations. Pre-treatment and recall periapical radiographs were evaluated and scored using the Periapical Index (PAI).

**Results:**

Autism and Attention deficit hyperactivity disorder were 39.4% of patients treated. Most treatments delivered were primary nonsurgical root canal treatment (95%). Warm vertical compaction of gutta-percha for obturation accounted for 88% of cases treated. Single cone obturation technique was used in 9 cases (12%) utilizing tricalcium silicate sealer. At the recall visits, 98.7% of teeth treated had survived. Twelve teeth have pre-treatment periapical lesions, and all healed except one. Female patients were found to have higher scores of PAI compared to male patients (23.7% vs 7.5%), yet insignificant. Only 10 patients with nonsurgical root canal treatment reported recurrent caries.

**Conclusion:**

This study demonstrates a high survival rate for nonsurgical endodontic treatment performed under GA in a cohort of patients with SHCNs. Interestingly, patients with social and communication disorders received the highest proportion of treatments under GA. These findings highlight the potential of GA-facilitated endodontics for this population. However, further research is warranted to explore additional methods for optimizing oral health outcomes in SHCNs.

## Introduction

An estimated 15% of the global population, or roughly 1 billion individuals, live with some form of disability. Among this group, 2–4% experience significant functional limitations, often encountering barriers to essential services, including oral healthcare [1]. The American Academy of Pediatric Dentistry (AAPD) defines Special Health Care Needs (SHCNs) as a broad spectrum of physical, developmental, mental, sensory, behavioral, cognitive, or emotional impairments requiring medical management, healthcare intervention, and specialized services or programs [2]. Examples of SHCNs include genetic syndromes [3], autism spectrum disorder (ASD) [4], and attention deficit hyperactivity disorder (ADHD) [5]. These conditions can significantly complicate dental care for the patient and the dental professional. Patients with SHCNs may also have complex dental needs arising from underlying medical conditions.

Pediatric dentists are often the primary providers in treating patients with special health care needs (SHCNs) since behavioral management is within the scope of their practice. Special care dentistry (SCD), formally recognized by the General Dental Council (GDC) in 2008, caters to individuals and groups with physical, sensory, intellectual, mental, medical, emotional, or social impairments or disabilities [6], necessitating specialized knowledge, skills, and equipment beyond those employed in general dentistry [7]. Endodontic treatment is a successful procedure and is preferred to preserve natural teeth. Due to the uncooperative behaviors often seen in such patients and the lengthy duration of endodontic procedures, most dentists recommended extracting permanent teeth requiring endodontic treatment rather than attempting endodontic treatment [8]. Meanwhile, nonsurgical root canal treatment (NSRCT) involves meticulous disinfection and shaping of the root canal system. Patient cooperation is required for optimal aseptic preparation, access, cleaning, and canal shaping. SHCNs undergoing NSRCT may face unique challenges, such as misusing these critical steps and potentially impacting treatment success [9].

General anesthesia (GA) is a facilitatory approach to providing high-quality dental care when chairside dental treatment under local anesthetic is impossible [10, 11]. Reports indicate improved oral health-related quality of life in SHCNs after dental treatment under GA [12]. Additionally, a 5-year survival rate estimate of 89.8% demonstrated that single-visit endodontic and restorative procedures under GA maintained sustained functionality of treated teeth [13]. The technical quality of the treatment is considered to be comparable with that performed under local anesthesia [14].

While previous research suggests promising success rates for endodontic treatment performed under GA [15], a fundamental knowledge gap exists regarding the specific patient population, treatment approaches, and long-term outcomes in this context. Existing studies often lack detailed information on the types of SHCNs represented, the materials employed, and the full range of treatment outcomes observed.

Thus, the present study aimed to provide a more comprehensive picture of nonsurgical endodontic treatment for SHCNs under GA. We conducted an observational cohort study, documenting the types of SHCNs treated, the specific procedures and materials used, and the treatment outcomes. This detailed analysis offers valuable insights into the feasibility and effectiveness of this approach for this patient population.

## Methods

### Study design and setting

The experimental design of this study has followed the Strengthening the Reporting of Observational Studies in Epidemiology (STROBE) guideline [16].

### Participants

A cross-sectional cohort clinical study was conducted. Patient records of patients who received endodontic treatment by residents on permanent teeth under GA between 2018 and 2022 were retrieved. SHCNs who received nonsurgical endodontic treatment and reported for the follow-up visit were included. The demographic and types of SHCNs of study participants were recorded.

### Treatment selection and procedures

All patients underwent an attempt at treatment under local anesthesia supplemented with nitrous oxide. Patients with significant cooperation difficulties were referred for treatment under GA. Diagnoses were established preoperatively based on a combination of patient-reported symptoms, clinical signs and symptoms, and periapical radiographic evaluation [17]. Due to the inherent challenges of obtaining reliable responses from some patients during clinical examinations, the accuracy of diagnostic tests used may have been limited in this study.

A standard nonsurgical endodontic treatment (NSRCT) was followed under GA protocol. In a few cases, vital pulp therapy was determined to be the appropriate treatment for teeth that exhibited pathological exposure during planned restorative procedures. All carious lesions were removed. The exposed pulp was covered with ProRoot MTA (Dentsply Sirona, Tulsa Dental, OK, USA) with a minimum thickness of 2 mm.

Following access cavity preparation for NSRCT, working length was established using an electronic apex locator (RootZX; J Morita, Irvine, CA). The root canals were then shaped and enlarged using a combination of engine-driven rotary instruments (ProTaper Gold; Dentsply Sirona, USA) and stainless-steel hand files (DiaDent, Cheongju, Korea). Throughout the procedure, irrigation was maintained with 5.25% sodium hypochlorite. Gutta-percha point obturation was performed using warm vertical compaction with various sealers. In a limited number of cases, where time constraints existed, the single cone technique was employed to achieve an adequate fill. For NSRC retreatment, gutta-percha was removed utilizing rotary file systems without solvent, followed by the abovementioned procedures. All procedures were conducted under rubber dam isolation.

### Outcome assessment

Teeth types, pulpal, and periapical diagnoses were extracted. The endodontic procedures, including treatment types and materials used, were categorized. For recall visits, one examiner performed a clinical examination whenever possible. Pain, extra- and intra-oral swelling, sinus tract, tenderness to percussion and palpation, probing depth, mobility, coronal restoration types, and caries were recorded. The periapical radiographs were obtained using a pro-X-ray unit [Planmeca Co., Helsinki, Finland] operating at 63 Kvp, 6 mA, and 0.160-sec exposure time. Following a paralleling technique, digitalized radiographs were obtained using a Trollbyte Plus film holder (Troll Dental, Sweden) and Planmeca Romexis® software at a focused object distance of 15 cm. All images were saved in TIFF format with a resolution of 300 dpi and dimensions of 4070 × 2947 pixels. For blinded evaluation, copies of the images were stored in separate folders for each rater. American Board-certified endodontists evaluated and scored Pre-treatment and recall radiographs using the Periapical Index (PAI) proposed by Orstavik et al [18]. The examiners were calibrated for intra-examiner reliability of the PAI scores before the study’s beginning by rescoring radiographs of 20 patients not involved in the study (*k* = 0.8). Radiographs were assessed in a quiet room using PowerPoint software after enhancement of the resolution as needed. Each observer scored the periapical area in each tooth studied.

Treatment outcomes were categorized based on the evaluation of the combined clinical and radiographic findings. Success was defined as the absence of clinical signs or symptoms and a PAI score of less than 3 (“Normal”). Teeth with a PAI score greater than 2 were classified as diseased. Additionally, the coronal restoration was assessed and categorized as either intact (any permanent restoration appearing intact clinically and radiographically) or recurrent caries (any permanent restoration with clinical or radiographic evidence of recurrent caries or open margins). Survival rate was also recorded, which is defined as the treated tooth remaining functional (not extracted).

#### Statistical data analysis

Descriptive statistics were used to summarize the characteristics of study participants and study outcomes. Numerical variables were summarized using mean, standard deviation, and range. Frequencies and proportions were used for categorical variables.

Inter-rater reliability for radiographic examination was assessed using weighted Kappa. Associations between the reason for admission, pulpal and periapical diagnosis, type of treatment, materials, intact margins, and recurrent caries were examined using crosstabulation tables and Fisher’s Exact test. Similarly, associations between treatment type, intact margins, and recurrent caries were examined using crosstabulation tables and Fisher’s Exact test. Fisher’s Exact test explores the association between two categorical variables when some frequency counts are low.

All inferential analysis was performed using the significance level 0.05, with *p*-values < 0.05 reported as statistically significant. Analysis was performed using IBM SPSS Statistics software (version 28).

## Results

### Demographic data and types of SHCNs included

**Fig. 1:**
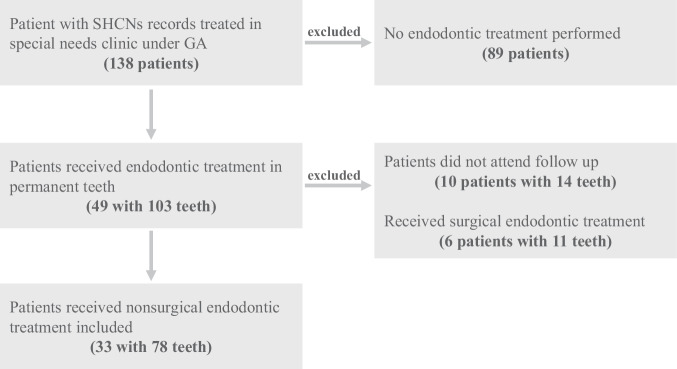
Flow chart of patients included and excluded.

Following an initial screening of 138 patients for eligibility, 105 were excluded due to (1) not receiving NSRCT (*n* = 89), (2) missing follow-up appointments (*n* = 10), or (3) undergoing surgical endodontic treatment (*n* = 6). Ultimately, 33 patients with 78 teeth satisfied all inclusion criteria and were included in the final analysis (Fig. 1). Demographic data and types of patients with SHCNs were summarized in Table 1. The patients had an average age of 20.48 years, with a nearly equal distribution of males (51.5%) and females (48.5%).

**Table 1:** Socio-demographic and medical characteristics of study participants.

Characteristics	Mean ± SD [Range] or Frequency (%)
Gender	
Male	17 (51.5%)
Female	16 (48.5%)
Age	20.48 ± 7.60 [10–46] years
Male	21.12 ± 8.48
Female	19.81 ± 6.75
Reason for admission	
Autism and Attention deficit hyperactivity	13 (39.4%)
Disorder (ADHD)	
Developmental delay	8 (24.2%)
Genetic disorders	6 (18.2%)
Neuromuscular disorders	4 (12.1%)
Neurological conditions	2 (6.1%)
Total number of patients	33

### Teeth types, diagnosis, endodontic treatment procedures, and materials used

Data was tabulated on tooth type, pulpal and periapical diagnoses, and administered treatments (Table 2). Most teeth treated were anterior (70.5%). The pulpal and periapical diagnoses were unidentified at 70.5% and 74.4%, respectively. Almost 95% of teeth received primary NSRCT. Tricalcium silicate sealer (Brasseler, Savannah, GA, USA) was used more frequently (88.6%) than other types. Warm vertical compaction of gutta-percha for obturation accounted for 88% of cases treated. Single cone obturation technique was used in 9 cases (12%) utilizing tricalcium silicate sealer. All treated teeth, except one (97.4%), received direct coronal restorations using 3M ESPE Composite (Filtek Z350 XT).

**Table 2:** Pulpal and periapical diagnoses, and treatment details.

Characteristics	Frequency (%)
Tooth type	
Anterior	55 (70.5%)
Premolar	12 (15.4%)
Molar	11 (14.1%)
Pulpal Dx	
Normal pulp	3 (3.8%)
Reversible pulpitis	6 (7.7%)
Asymptomatic irreversible pulpitis	5 (6.4%)
Symptomatic irreversible pulpitis	2 (2.6%)
Necrotic pulp	5 (6.4%)
Previously initiated	1 (1.3%)
Previously treated	1 (1.3%)
Unidentified	55 (70.5%)
Periapical Dx	
Normal Periapical Tissue	12 (15.4%)
Asymptomatic Apical Periodontitis	5 (6.4%)
Chronic Apical Abscess	3 (3.8%)
Unidentified	58 (74.4%)
Type of treatment	
Nonsurgical root canal treatment	74 (94.9%)
Nonsurgical root canal retreatment	1 (1.3%)
Vital pulp therapy	3 (3.8%)
Material	
AH Plus sealer	3 (3.8%)
Tricalcium silicate sealer	69 (88.6%)
Zinc oxide eugenol sealer	3 (3.8%)
Mineral trioxide aggregate	3 (3.8%)
Total number of teeth	78

The associations between the reason for admission, pulpal and periapical diagnosis, type of treatment, and sealer used were examined using crosstabulation tables and Fisher’s Exact test (Table 3). There was a statistically significant association found between admission reason and pulpal diagnosis (Fisher’s Exact test *p* < 0.001). More than a third of cases with genetic disorders (36.4%) had necrotic pulp. Most of the autism and ADHD, developmental delay, and neuromuscular disorders had an unidentified pulpal diagnosis (71.0%, 87.0%, and 66.7%, respectively). Autism and ADHD cases had reversible pulpitis (19.4%). Half of the neurological condition cases have asymptomatic irreversible pulpitis.

**Table 3:** Reason for admission associations with diagnoses, treatments, and materials used.

	Reason for admission				
	Autism and Attention deficit hyperactivity disorder (*n* = 31)	Developmental delay (*n* = 23)	Genetic disorders (*n* = 11)	Neuromuscular disorders (*n* = 9)	Neurological conditions (*n* = 4)
Pulpal Dx					
Normal pulp	0 (0%)	3 (13.0%)	0 (0%)	0 (0%)	0 (0%)
Reversible pulpitis	6 (19.4%)	0 (0%)	0 (0%)	0 (0%)	0 (0%)
Asymptomatic irreversible pulpitis	0 (0%)	0 (0%)	2 (18.2%)	1 (11.1%)	2 (50.0%)
Symptomatic irreversible pulpitis	2 (6.5%)	0 (0%)	0 (0%)	0 (0%)	0 (0%)
Necrotic pulp	1 (3.1%)	0 (0%)	4 (36.4%)	0 (0%)	0 (0%)
Previously initiated	0 (0%)	0 (0%)	0 (0%)	1 (11.1%)	0 (0%)
Previously treated	0 (0%)	0 (0%)	0 (0%)	1 (11.1%)	0 (0%)
Unidentified	22 (71.0%)	20 (87.0%)	5 (45.4%)	6 (66.7%)	2 (50.0%)
Periapical Dx					
Normal Periapical Tissue	5 (16.1%)	3 (13.0%)	0 (0%)	2 (22.2%)	2 (50.0%)
Asymptomatic Apical Periodontitis	2 (6.5%)	0 (0%)	3 (27.3%)	0 (0%)	0 (0%)
Chronic Apical Abscess	2 (6.5%)	0 (0%)	1 (9.1%)	0 (0%)	0 (0%)
Unidentified	22 (71.0%)	20 (87.0%)	7 (63.6%)	7 (77.8%)	2 (50.0%)
Type of treatment					
Nonsurgical root canal treatment	30 (96.8%)	23 (100%)	9 (81.8%)	8 (88.9%)	4 (100%)
Nonsurgical root canal retreatment	0 (0%)	0 (0%)	0 (0%)	1 (11.1%)	0 (0%)
Vital pulp therapy	1 (3.2%)	0 (0%)	2 (18.2%)	0 (0%)	0 (0%)
Material					
AH Plus sealer	1 (3.2%)	2 (8.7%)	0 (0%)	0 (0%)	0 (0%)
Tricalcium silicate sealer	28 (90.4%)	21 (91.3%)	7 (63.6%)	9 (100%)	4 (100%)
Zinc oxide eugenol sealer	1 (3.2%)	0 (0%)	2 (18.2%)	0 (0%)	0 (0%)
Mineral trioxide aggregate	1 (3.2%)	0 (0%)	2 (18.2%)	0 (0%)	0 (0%)

#### Clinical outcomes and survival rates

All patients included in the study attended the recall visit. All teeth treated, except one, were present (98.7% survival rate) and were available to be clinically examined. The recall period ranged between 3 and 39 months, with an average of 20.32 months (median = 21 months). Most cases (70.5%) have a follow-up period over 12 months.

The recall clinical examination information is summarized in Table 4. Coronal restoration integrity and caries development were assessed to evaluate the potential risk types of patients with SHCNs. Patients with neuromuscular (66.7%) and neurological conditions (100%) exhibited a higher prevalence of intact coronal restoration. However, no statistically significant association was found between the reason for admission and recurrent caries (Fisher’s Exact test, *p* = 0.064), and less than 25% of all patients presented with recurrent caries. (Table 5).

**Table 4:** Recall clinical examination details.

Characteristics	Frequency (%)
Survival	
No	1 (1.3%)
Yes	77 (98.7%)
Percussion	
No	9 (11.6%)
Yes	3 (3.8%)
Unknown	66 (84.6%)
Palpation	
No	12 (15.4%)
Yes	0 (0%)
Unknown	66 (84.6%)
Mobility	
No mobility	71 (91.1%)
Grade 1	4 (5.1%)
Unknown	3 (3.8%)
Sinus tract	
No	78 (100%)
Yes	0 (0%)
Probing	
Less than 3 mm	63 (80.8%)
Unknown	15 (19.2%)
Intraoral swelling	
No	78 (100%)
Yes	0 (0%)
Extraoral swelling	
No	78 (100%)
Yes	0 (0%)
Coronal restoration	
Direct	76 (97.4%)
Full coverage	1 (1.3%)
Intact margins	
No	12 (15.4%)
Yes	65 (83.3%)
Recurrent caries	
No	67 (85.9%)
Yes	10 (12.8%)

**Table 5:** Reasons for admission associations with intact coronal restoration margins and recurrent caries.

	Reason for admission				
	Autism and Attention deficit hyperactivity disorder (*n* = 31)	Developmental delay (*n* = 23)	Genetic disorders (*n* = 11)	Neuromuscular disorders (*n* = 9)	Neurological conditions (*n* = 4)
Intact margins	23 (74.2%)	22 (95.7%)	10 (90.9%)	6 (66.7%)	4 (100%)
Recurrent caries	8 (25.8%)	1 (4.3%)	0 (0%)	1 (11.1%)	0 (0%)

Furthermore, the associations between treatment type, intact coronal restoration margins, and recurrent caries were examined using crosstabulation tables and Fisher’s Exact test. No significant difference was found between treatment types regarding intact coronal restoration margins (Fisher’s Exact test *p* = 1.00). Within each treatment type, most patients have intact coronal restoration margins. Also, no significant difference was found between treatment types regarding recurrent caries (Fisher’s Exact test *p* = 1.00). Only 10 patients with NSRCT reported recurrent caries.

#### Analysis of PAI Scores

Two board-certified endodontists performed a radiographic examination. Inter-examiner reliability for pre-, recall, and PAI outcomes was achieved using Cohen’s Kappa. At the recall visit, 9 patients did not have periapical radiographs due to behavioral difficulties and were excluded from the reliability analysis. The 138 measurements were performed by two raters who agreed on 125 assessments (91% agreement rate). As the measurements can be considered on an ordinal scale, Cohen’s weighted Kappa would be a more appropriate measure of agreement between rater s [19]. If two raters disagree by 1 point (1 vs. 2 or 2 vs. 3), it represents less disagreement than raters differing by 3 points (1 vs. 4 or 2 vs. 5). Thus, the weighted Kappa was 0.692 (95% CI 0.578–0.808), indicating substantial agreement.

The following analysis was based on scores provided by one examiner. Associations between the reason for admission, gender, and pre-treatment radiographic examination were examined using crosstabulation tables and Fisher’s Exact test. A score of 3 or more was reported only among patients with the following three reasons for admission: autism and attention deficit hyperactivity disorder (19.4%), developmental delay (17.4%), and genetic disorders (18.2%). However, the association between the reason for admission and pre-radiographic examination was not statistically significant, Fisher’s Exact test *p* = 0.303. Female patients were found to have a higher score of 3 or more compared to male patients (23.7% vs. 7.5%). However, the difference was not statistically significant (only approaches/borderline significant), Fisher’s Exact test *p* = 0.053.

The above analysis showed 12 records with 3 or more scores at pre-assessment. Out of these 12 records, 11 were found normal (1 or 2) at the recall visit. The analysis below focused on these 11 cases. Associations between recall PAI, intact coronal restoration margins, and recurrent caries were examined using crosstabulation tables and Fisher’s Exact test (Table 6).

**Table 6:** Recall PAI associations with coronal restoration

	Recall PAI rater 1		
	Unidentified [code 0] (*n* = 9)	Normal [code 1–2] (*n* = 66)	Disease [code 3–5] (*n* = 3)
Intact margins	7 (77.8%)	56 (84.8%)	2 (66.7%)
Recurrent caries	0 (0%)	9 (13.6%)	1 (33.3%)
	**Recall PAI rater 2**		
	**Unidentified [code 0] (*n*** = **14)**	**Normal [code 1–2] (*n*** = **61)**	**Disease [code 3–5] (*n*** = **3)**
Intact margins	12 (85.7%)	51 (83.6%)	2 (66.7%)
Recurrent caries	0 (0%)	9 (14.8%)	1 (33.3%)

No significant difference was found between recall PAI regarding intact coronal restoration margins (Fisher’s Exact test *p* = 0.61). Also, no significant difference was found between recall PAI regarding recurrent caries (Fisher’s Exact test *p* = 0.27).

## Discussion

Building upon prior research analyzing endodontic treatment outcomes in patients with disabilities [13, 15], the present study primarily focused on documenting the types of SHCNs treated, the specific procedures and materials used, and the treatment outcomes for permanent teeth. The findings demonstrated a high success rate, with 98% of treated teeth surviving at follow-up visits ranging from 3 to 39 months. The high success rate could be attributed to several factors related to improved treatment quality. A previous study evaluated the quality of endodontic treatment under GA in patients with special needs (255 teeth) compared to treatments under local anesthesia (LA) (264 teeth). Across both groups, 63% of cases achieved a satisfactory quality [14].

It is important to acknowledge that recent studies have reported varying success rates. For instance, a retrospective study found an 87.6% survival rate for 280 RCTs performed under GA on patients with SHCNs over a nine-year follow-up period [20]. Several factors might explain this discrepancy. Firstly, the current study involved a higher proportion of treated anterior teeth, which are less complex than posterior teeth. Additionally, the shorter follow-up period in our study might underestimate potential long-term complications.

Traditionally, endodontic treatment success is assessed based on the functionality and survival of treated teeth alongside healthy pulpal and periapical tissues [21]. Pre-existing periapical lesions are established risk factors for treatment outcomes [22–24]. Notably, in the present study, only 12 cases (15.3%) presented with such lesions. While another study reported a higher proportion of cases (25.8%) with preoperative apical resorption [15], The present study achieved high success rates. Only one case with a pre-existing lesion did not heal at the follow-up visit. This success rate surpasses those reported in previous studies on teeth with periapical lesions (outcomes: 78–85%) [25]. Studies on SHCNs have reported a complete periapical healing of 81–87% for endodontically treated teeth under GA [15, 26]. However, it is essential to acknowledge a potential limitation. The small subset (15.3%, *n* = 12) of treated teeth with pre-existing periapical lesions in the present study might represent less complex cases at baseline, potentially contributing to the observed high success rate. Despite this limitation, these results suggest that endodontic treatment should be prioritized to preserve restorable teeth, even in teeth with periapical lesions. Furthermore, previous research has shown that such treatment can significantly improve caregiver-reported oral health-related quality of life [12].

It is known that endodontic diagnosis is mandatory prior RCT [27]. Patient cooperation limitations due to SHCNs hindered the analysis of preoperative and endodontic prognostic factors in the present study. Consequently, the pulpal and periapical diagnoses were often not definitively established. It agrees with previous research that reported difficulties in performing comprehensive clinical examinations and recording potential risk factors for treatment success in patients with SHCNs [28, 29]. These limitations can necessitate intraoperative decision-making by clinicians regarding the most appropriate endodontic treatment approach.

In the present study, 76 of the 78 treated cases (97.4%) received direct coronal restorations following endodontic treatment. Limitations associated with the GA setting and the age of the patients might have restricted the use of full coverage restorations. Core build-up and direct restorations may be more feasible options in such circumstances. While direct restorations offer a viable treatment option, it is crucial to acknowledge their potential limitations compared to full crowns. A previous study reported higher survival rates for endodontically treated teeth restored with full crowns (99.2% at 3 years) than direct restorations (92.8% at 3 years). This difference persisted at the 5-year follow-up (95.5% vs. 84.6%) [13]. In the present study, some teeth experienced coronal fractures with direct restorations, suggesting a potential for increased fracture rates over time compared to full crowns.

In this study, we investigated the reasons for admitting patients for dental treatment under GA. Among the patients included, 39.4% had diagnoses of autism and/or ADHD. This finding aligns with previous research demonstrating a high prevalence of caries in autistic populations [4]. Furthermore, a similar study reported an association between autism and ADHD with the need for endodontic treatment under GA [20]. As recent studies continue to report an increase in the prevalence of social and communication disorders over time, further research is needed to explore methods to benefit their optimum oral health [30].

Most endodontic treatments in the present study were primary NSRCT (94. 9%). Tricalcium silicate sealers were utilized in 88.6%. Recent studies advocate using calcium silicate cement-based sealers due to their excellent physiochemical and biological characteristics, and clinical success [31–33]. One advantage is that it allows the clinician to utilize traditional obturation techniques, lateral and warm vertical compaction, and single cone technique. The present study’s high success and survival rate support the use of calcium silicate sealer for obturation.

The present study has several limitations inherent to its observational design. Firstly, like most observational studies, the potential for confounding factors exists. While several preoperative and prognostic factors were analyzed and found statistically insignificant, other unmeasured variables could have influenced the outcomes. Secondly, using GA in some patients limited the ability to perform a comprehensive preoperative examination and establish a definitive diagnosis. Additionally, endodontic procedures were modified under GA to minimize treatment time and facilitate other procedures. Furthermore, the study included a heterogeneous population of patients with SHCNs without stratifying for underlying medical conditions, which might have impacted treatment outcomes. Finally, the inclusion of all teeth types with varying root development stages introduced additional variability. These limitations collectively raise the possibility of underestimating or overestimating the treatment success rates reported.

## Conclusion

This study underscores the efficacy of combining GA with contemporary nonsurgical endodontic procedures for patients with SHCNs. The high survival rate, successful resolution of periapical lesions, and minimal recurrent caries observed in this study suggest that GA can be a valuable tool to facilitate successful endodontic treatment, improving oral health outcomes in this patient population.

## Data Availability

The data that support the findings of this study are available from the corresponding author, Fahd Alsalleeh upon request.
